# Conjunctival Scarring in Trachoma Is Associated with the HLA-C Ligand of KIR and Is Exacerbated by Heterozygosity at KIR2DL2/KIR2DL3

**DOI:** 10.1371/journal.pntd.0002744

**Published:** 2014-03-20

**Authors:** Chrissy h. Roberts, Sandra Molina, Pateh Makalo, Hassan Joof, Emma M. Harding-Esch, Sarah E. Burr, David C. W. Mabey, Robin L. Bailey, Matthew J. Burton, Martin J. Holland

**Affiliations:** 1 Clinical Research Department, London School of Hygiene and Tropical Medicine, London, United Kingdom; 2 Medical Research Council Unit, The Gambia, Atlantic Boulevard, Fajara, The Gambia; 3 International Centre for Eye Health, London School of Hygiene and Tropical Medicine, London, United Kingdom; National Institutes of Health, United States of America

## Abstract

**Background:**

*Chlamydia trachomatis* is globally the predominant infectious cause of blindness and one of the most common bacterial causes of sexually transmitted infection. Infections of the conjunctiva cause the blinding disease trachoma, an immuno-pathological disease that is characterised by chronic conjunctival inflammation and fibrosis. The polymorphic Killer-cell Immunoglobulin-like Receptors (KIR) are found on Natural Killer cells and have co-evolved with the Human Leucocyte Antigen (HLA) class I system. Certain genetic constellations of KIR and HLA class I polymorphisms are associated with a number of diseases in which modulation of the innate responses to viral and intracellular bacterial pathogens is central.

**Methodology:**

A sample of 134 Gambian pedigrees selected to contain at least one individual with conjunctival scarring in the F_1_ generation was used. Individuals (n = 830) were genotyped for HLA class I and KIR gene families. Family Based Association Tests and Case Pseudo-control tests were used to extend tests for transmission disequilibrium to take full advantage of the family design, genetic model and phenotype.

**Principle findings:**

We found that the odds of trachomatous scarring increased with the number of genome copies of HLA-C2 (C1/C2 OR = 2.29 _BH_P-value = 0.006; C2/C2 OR = 3.97 _BH_P-value = 0.0004) and further increased when both *KIR2DL2* and *KIR2DL3* (C2/C2 OR = 5.95 _BH_P-value = 0.006) were present.

**Conclusions:**

To explain the observations in the context of chlamydial infection and trachoma we propose a two-stage model of response and disease that balances the cytolytic response of KIR expressing NK cells with the ability to secrete interferon gamma, a combination that may cause pathology. The data presented indicate that HLA-C genotypes are important determinants of conjunctival scarring in trachoma and that *KIR2DL2*/*KIR2DL3* heterozygosity further increases risk of conjunctival scarring in individuals carrying HLA-C2.

## Author Summary

*Chlamydia trachomatis* is a pathogen that causes sexually transmitted infections (STIs) and the blinding disease trachoma. Natural Killer (NK) cells are part of the host immune system's first line of defence against infection. NK cell functions are genetically encoded and differences between individuals mean that some people are better able to respond to infections than others. We found that in certain combinations, specific variants of the gene *HLA-C* (Human Leucocyte Antigen, C) and of a complex set of genes called the Killer-cell Immunoglobulin-like Receptors (KIR) were associated with a six-fold increase in the relative risk of scarring tissue damage resulting from ocular *C. trachomatis* infection (trachoma). This combination of genetic variants may reduce the host's ability to effectively resolve infections and result in a harmful immune response that ultimately leads to tissue damage and scarring. KIR^+^ NK cells are potential cellular mediators of the damaging immune response. Previous studies have identified that the same HLA-KIR genetic constellation that associates with trachoma is actually protective against infectious diseases such as malaria and tuberculosis. The high frequency of the trachoma-associated constellation in African populations may therefore be explained by the evolutionary benefits of protection from the complications of severe disease.

## Introduction

*Chlamydia trachomatis* (*Ct*) is an obligate intracellular bacterium [1] which causes significant morbidity as the causative factor of around 106 million new sexually transmitted infections per annum [2]. As the cause of trachoma, the same bacterium is the most common infectious cause of blindness [3]. *Ct* serovars exhibit highly specific tissue tropism, with serovars A–C being limited to the mucosal epithelium of the ocular conjunctiva. The remaining serovars are sexually transmitted, but whilst serovars D–K are limited to the mucosal epithelia of the genitourinary tract and rectum, the strains L1–L3 are able to invade other tissues including the lymph nodes. Ocular infection in trachoma is spread among young persons through exposure to secretions from the infected eye via direct physical contact, on fomites or by eye-seeking flies [4]. Repeated and prolonged cycles of infection and inflammation have been identified as the main factors that lead to the progressive formation of fibrotic scars on the tarsal conjunctiva, which ultimately becomes deformed. This can cause entropion and trachomatous trichiasis (TT), a condition where the eyelashes turn inwards and irreversibly damage the cornea by scratching the globe of the eye. If left unchecked, TT causes corneal opacity, visual impairment and blindness.

Active trachoma is frequently found in the absence of detectable *Ct* infection and both tissue damage and scarring are thought to be the result of a chronic immuno-pathological reaction [5]. Human conjunctival transcriptome studies in trachoma suggest that in addition to T cell and innate responses of epithelial cells, the activation and cytotoxic responses of natural killer (NK) cells is an important determinant of the severity of active trachoma [6], [7]. NK cells are a rich source of multiple chemokines and cytokines, including interferon gamma (IFNγ), a cytokine that is central to the control of chlamydial intracellular development and growth. IFNγ also has anti-fibrotic properties that can counteract the effects of TGF-β and inhibit fibroblast proliferation and collagen synthesis [8], but when inappropriately expressed may cause immunopathology. NK cells in mucosal-associated lymphoid tissues are known to be important in the maintenance of epithelial cell integrity via production of the cytokine IL-22 [9]. NK cells therefore have the potential to fulfil multiple roles that encompass tissue homeostasis, tissue re-modelling and immunity.

Early studies in murine chlamydial model infections found that NK cell depletion exacerbated disease, delayed clearance and limited the development of specific T cell responses [10], [11]. Subsequent studies have confirmed that in response to chlamydial stimulation, NK cells are promoters of T cell immunity and a major source of IFNγ [10], [12] but their role as lytic effector cells is less clear. Although *Ct* infected cell lines are lysed *in vitro*, NK cells purified from the peripheral blood of individuals with current chlamydial infection had diminished lytic activity (and reduced IFNγ) compared with uninfected controls [13]. Population diversity in the highly polymorphic genes that encode the variable NK receptors and their ligands [14] along with functional heterogeneity in the NK cell repertoire may account for these findings [15].

Trachoma is a complex inflammatory fibrotic disease in which host polymorphism in immune response genes plays a significant role [16]–[18]. The conjunctival epithelial surface is compromised in trachoma [5] as a result of the host response to the causative bacterium, which occupies an intracellular niche. Therefore the mechanisms used by NK cells in the control of other intracellular infections such as Hepatitis B [19], Hepatitis C [20] and HIV [21]–[23] might also be effective against intracellular *Ct*.

NK cells become activated when they are released from inhibition that is normally bound by interaction of specific HLA class I ligands with inhibitory Killer-cell Immunoglobulin-like Receptors (KIRs) [24]. The ligands of several inhibitory KIR have been described including HLA-A3 and HLA-A11 alleles, which are ligands of the KIR3DL2 receptor [25], [26] and the HLA-Bw4 public epitope which is the ligand of KIR3DL1 [27], [28]. *HLA-C* alleles can be classified (according to a functional dimorphism at amino acid position 80) as carrying one of two KIR binding epitopes, which are known as HLA-C1 and HLA-C2 [29]. The HLA-C2 group of alleles (HLA-C*02/04/05/06…) are ligands of the inhibitory receptor KIR2DL1 [30]–[32] and its activating counterpart KIR2DS1 [33]. The HLA-C1 group alleles (HLA-Cw*01/03/07/08…) are ligands of both KIR2DL2 and KIR2DL3 [30]–[32], however, the latter KIR are both able to cross-react (with differing avidities) with a small number of HLA-C2 and HLA-B allotypes [34]. Although germ-line encoded, the KIR gene system is highly polymorphic and exhibits extensive diversity both between individuals and between populations [35]–[38]. KIRs exhibit haplotype diversity such that different individuals possess variable gene contents. Since KIR and HLA are also found on different chromosomes, individuals can possess a KIR for which they have no cognate ligand, or vice versa. The extensive polymorphism in the KIR system culminates in a repertoire of NK cells within an individual that is more or less sensitive to release from inhibition under appropriate physiological conditions [39]. The strength of the signals mediated by interactions between specific HLA and KIR alleles is also highly variable [29], [40], [41] and this further limits overall NK cell responsiveness [42]. In part the responsiveness might be predicted by the presence of type ‘A’ and type ‘B’ KIR haplotypes. Type A haplotypes carry genes encoding predominantly inhibitory KIRs. B haplotypes contain some or all of the same genes found on A haplotypes, but additionally may carry the inhibitory *KIR2DL2* and *KIR2DL5* genes and numerous activating KIRs [36]. KIR haplotypes can be separated in to two variable regions, defined by their orientation towards the centromeric (Cen) or telomeric (Tel) regions of the chromosome [43]. The KIR A and B haplotypes are present in all populations studied to date and are thought to be maintained by the balancing selection pressures of infection, immunopathology and healthy reproduction [44]–[47]. In recent human history, a wide range of infectious diseases may have reduced the balancing effects in African populations, leading to more directional selection and a unique pattern of HLA and KIR diversity in this region [38], [47]. We therefore assessed the extent to which host genotypes at the HLA and KIR loci were associated with trachomatous scarring in a trachoma endemic population from The Gambia.

## Methods

### Ethics statement

The study was conducted in accordance with the tenets of the Declaration of Helsinki. The Ethics Committee of the Gambian Government/Medical Research Council Unit, and the ethics committee of the London School of Hygiene and Tropical Medicine approved the study (MRC SCC1177). Individual written informed consent was obtained from all adult participants. Written informed consent was obtained from a parent/guardian on behalf of those subjects aged <18 years who wished to take part in the study. All samples were anonymised.

### Study population, sampling and ascertainment

We selected a family study design and identified probands at a relatively early age for clinical signs of conjunctival scarring. This maximised statistical power whilst controlling for population stratification through the use of related control samples. The study population came from multiple regions of The Gambia and included multiplex families of mixed ethnic background. Families were ascertained through the identification of probands in which there were signs of trachomatous scarring at an early age (age ≤30 years). This approach maximised the extent to which genetic rather than environmental factors could be expected to have contributed significantly to the probands' phenotypes. We recruited first-degree relations of the probands. In most cases this meant that we sampled both biological parents of the probands and all their (self-described) full siblings. Samples for DNA analysis were collected from buccal mucosae using sterile cyto-brushes (Part Number F-440151, SLS, Nottingham, UK). After collection, brushes were returned to their original packaging and stored dry at room temperature for up to 6 months [48] before DNA extraction was performed using a salting out procedure.

### Sample size and power calculation

An average of 4 offspring per family was assumed with a population prevalence of scarring in those <30 years of age in The Gambia of ∼2% [49]. The Pedigree Based Association Test (PBAT) v3.6 program [50] was used to calculate the power of the study to detect with 95% confidence (α<0.05) a genetic association with odds ratios 1.5, 2 and 3 when the hypothetical disease allele had a frequency between 0.01 and 0.50. Figure S1 shows the estimated power of this study to detect genetic associations with trachomatous scarring at a range of allele frequencies and effect magnitudes, given the sample size. We had >90% power to detect an effect size greater than an odds ratio (OR) = 3 when the allele frequency was ≥0.05 and similar power to detect an effect size of OR = 2 when the allele frequency was ≥0.19.

### Trachoma phenotypes

Trachoma was graded in the field using the WHO simplified grading system by field supervisors certified for trachoma grading with regular performance checks as described by the PRET clinical trial manual of operations [51]. Left and right tarsal conjunctivae of all subjects were photographed as described by Derrick *et al.* [52]. Photographs were subsequently reviewed by two ophthalmologists with experience of grading trachoma and a final grade agreed. Subjects were assigned to the ‘scarred’ group if there were any signs of trachomatous scarring, in either eye. Individuals where phenotypes could not be confirmed for reasons of poor quality photography (n = 5) did not contribute to the statistical tests of association.

### Genotyping and pedigree tests for Mendelian inheritance

KIR genotyping for the presence or absence of 17 KIR genes was performed by PCR using the set of sequence specific primers described by Vilches *et. al.* [53]. The genotyping method was validated by participation in the UCLA Immunogenetics Center KIR exchange programme (http://www.hla.ucla.edu/cellDna.htm). Medium resolution *HLA-A*, *-B* and *-C* genotyping was performed using LABtype sequence specific oligonucleotide probes (OneLambda, Canoga Park, CA. USA) on a Luminex platform (Luminexcorp, Austin, TX. USA). Medium resolution HLA typing data generates strings of possible allele combinations. Information from the HLA genotypes of family members was used to reduce the length of the strings of possible allele pairs and to eliminate alleles that were not compatible with Mendelian inheritance within a given pedigree. Strings were further shortened where possible to include only common and well-defined alleles [54]. In order to maximise statistical power, highly sequence similar HLA alleles were combined in to groups (table S1) before FBAT. KIR ligands of HLA (HLA-A*03/11/Bw4, *HLA-B*-Bw4, *HLA-C*1/*C*2) were inferred from the full HLA genotypes of individual specimens rather than the reduced strings. The HLA-C*16:01 (HLA-C1) and HLA-C*16:02 (HLA-C2) alleles were frequently ambiguous and where this was the case alleles were assigned to HLA-C*16:01 because the HLA-C*16:02 allele has not been observed in other West African populations whilst HLA-C*16:01 is very common (data from allelefrequencies.net). HLA types were used to identify cases of parental mis-assignment and inconsistent parent-offspring genotypes. KIR phenotypes (presence/absence) were tested for Mendelian inconsistencies. *KIR2DL5*, *KIR2DS3* and *KIR2DS5* were not included in the association tests as they can segregate to both Cen-B and Tel-B regions and confound haplotype assignments.

### Statistical analysis

KIR gene frequencies were compared to those of other world populations using data from allelefrequencies.net and PCA using R. Family based tests of HLA association were carried out using FBAT v.2.0.3 [55] performing a series of bi-allelic tests (i.e. association of an index allele against all other alleles) under an additive genetic model and the null hypothesis of no linkage and no association of any factor of the HLA system with trachomatous scarring. This approach is robust to effects of population structure [55], [56] and is applicable to a data set with samples originating in mixed ethnic backgrounds. We tested for associations between scarring and all HLA alleles with a sample frequency greater than 0.05 with an offset value of 0.02 (population prevalence of scarring in persons ≤30 years of age) to allow the unaffected siblings to contribute to the test statistic. All FBAT p-values were adjusted using a conservative Bonferroni correction. Significant associations were tested again using a case/pseudo-control conditional logistic regression (CLR) [57], which generated estimates of odds ratios and associated p-values. To test for independence between the disease-associated alleles, we included all alleles that had a corrected p<0.05 in a multivariate CLR model. To establish whether significant HLA associations were restricted to F_1_ subjects with specific KIR genotypes we tested the full data set under a genotype model [58], [59], using CLR, in different subsets of the F_1_ data where the population was limited by the KIR genotype. Because of the high linkage disequilibrium between factors of the KIR system, these tests were not considered to be independent and test statistics were corrected using the Benjamini-Hochberg method.

## Results

### Sample population

We sampled 830 individuals from 134 pedigrees and 146 nuclear families in which scarring trachoma had been identified in the first filial (F_1_) generation. The self-described ethnic background of the parental (P_0_) population (n = 260) was approximately 40% Mandinka, 23% Fula, 15% Jola, 15% Wolof, 5% Bambara and 2% other minority ethnic groups. There were 570 persons in the F_1_ generation, where the gender distribution was 52% (n = 296) male and 48% (n = 274) female. The median number of offspring per pedigree was 4 (range 1–11). Eight families had one missing parent. There were 180 (∼32%) cases of trachomatous scarring in the F_1_ generation and of these, 72 (40%) were female and 108 (60%) were male. Three hundred and eighty six (∼67.8%) F_1_ individuals were unaffected and phenotypic status could not be confirmed for 4 (<1%). Table 1 gives a detailed description of the phenotype distribution in the families. Detailed examination of photographs revealed that 12 probands did not have sufficient signs of trachomatous scarring. One proband could not be graded. In all the families where there was no photography confirmed scarred proband, at least one sibling was identified who was under 30 years of age and had signs of scarring. HLA genotyping identified paternal misassignment in 63 F_1_ individuals (11%) who were reassigned to an unknown father but were otherwise retained for analysis.

**Table 1:** Clinical and Demographic features of the sample.

		Scarring (C) Grade (n [%])				
Group	Age in years (Median [min - max])	C0	C1	C2	C3	No FPC Grading
**F_1_ Generation**						
Total (n = 570)	08 [0.1–40]	386 (67.8)	*48 (8.4)*	112 (19.6)	20 (3.5)	4 (0.7)
Male (n = 296)	08 [0.1–28]	188 (63.6)	*25 (8.4)*	71 (24.0)	12 (4.0)	0 (0.0)
Female (n = 274)	08 [0.1–40]	198 (72.2)	*23 (8.4)*	41 (15.0)	8 (2.9)	4 (1.5)
Probands (n = 134)	05 [0.3–22]	12 (9.0)	*12 (9.0)*	93 (69.4)	16 (11.9)	1 (0.7)
Siblings (n = 436)	10 [0.1–40]	374 (85.7)	*36 (8.3)*	19 (4.4)	4 (0.9)	3 (0.7)
**P_0_ Generation**						
Total (n = 260)	39 [18–72]	121 (46.6)	*65 (25.0)*	69 (26.5)	4 (1.5)	1 (0.4)
Female (n = 132)	34 [18–65]	77 (58.2)	*22 (16.7)*	31 (23.5)	1 (0.8)	1 (0.8)
Male (n = 128)	45 [23–72]	44 (34.5)	*43 (33.6)*	38 (29.7)	3 (2.3)	0 (0.0)
**Families**	Minimum	1^st^ Quantile	Median	3^rd^ Quantile	Maximum	
Number persons F_1_	1	3	4	5	11	

### HLA and KIR genotypes

**Figure 1:**
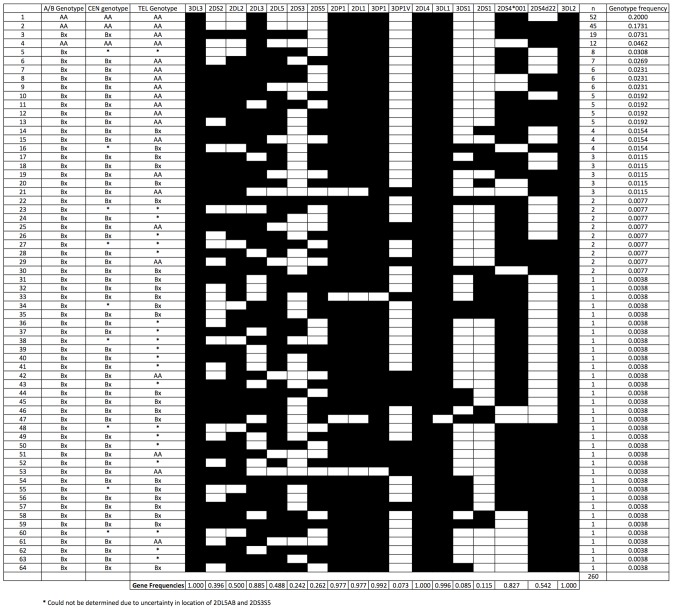
KIR genotypes and observed frequencies in the P_0_ population (n = 260).

**Figure 2:**
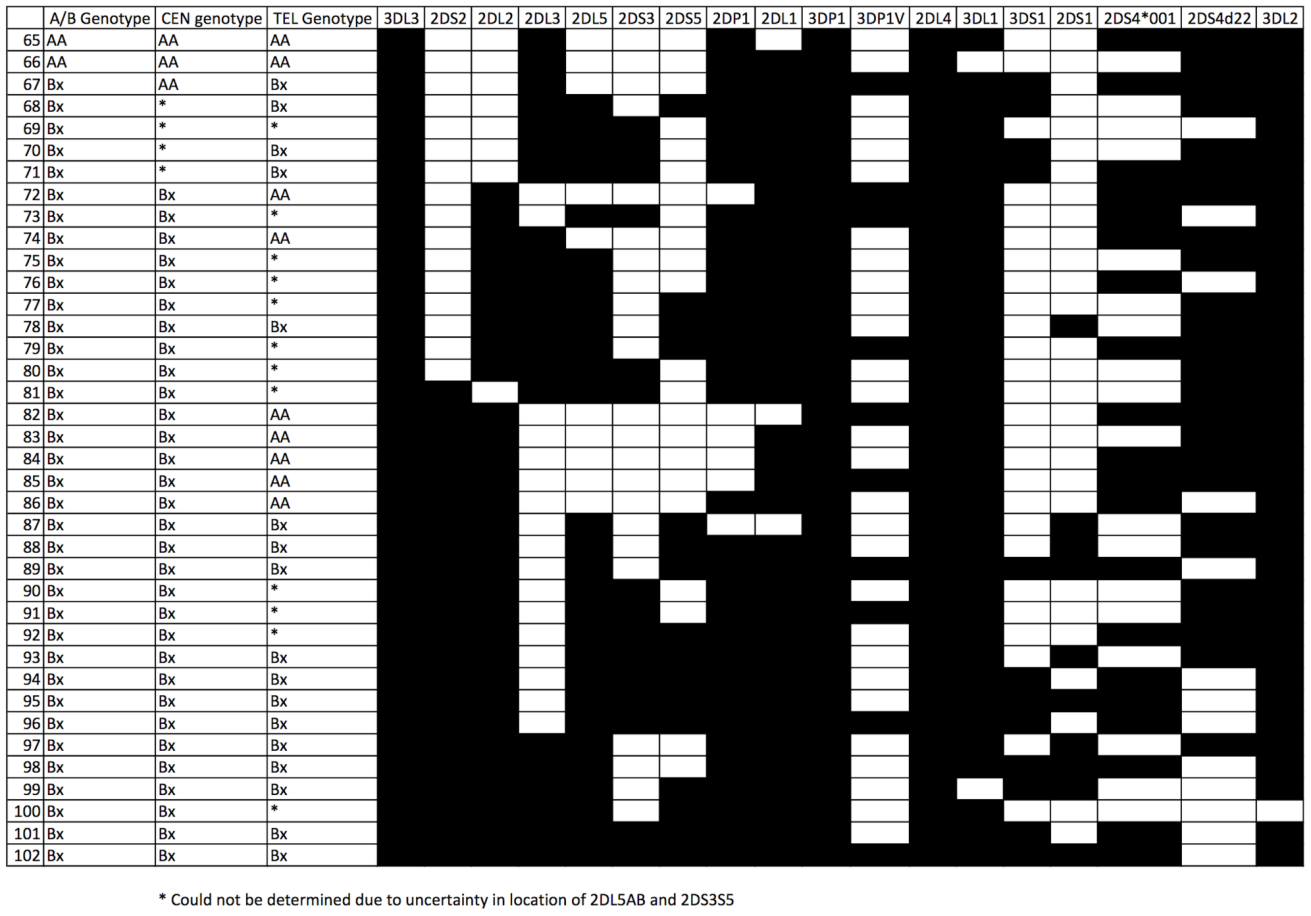
KIR genotypes and that were uniquely identified in the F_1_ population.

Table 2 shows the Family Based Association Test (FBAT) estimates of the HLA allele and KIR epitope frequencies in the sample population. Figure 1 describes the 64 unique KIR genotypes that were observed in the P_0_ generation. Thirty-eight additional KIR genotypes were revealed by re-assortment of the parental haplotypes in the F_1_ generation (Figure 2). All observed genotypes were assigned as either the ‘AA’ or ‘Bx’ genotypes (where Bx includes both AB and BB genotypes) for the full KIR region and where possible, for each of the Cen and Tel regions. A number of unusual genotypes were identified in this population, most notably, 10.4% of P_0_ individuals (n = 27/260) possessed *KIR2DL2* but not *KIR2DS2*.

**Table 2:** HLA Class I allele frequencies and FBAT tests of association.

Locus	Allele Name*	Allele frequency (P_0_)	# informative families (n)	S-E(S)	Var(S)	Z	P	Corrected P
*HLA-A*	A*02:01	0.067	37	−4.66	10.767	−1.42	0.155552	1
	A*23:01	0.164	67	−0.56	24.675	−0.113	0.910241	1
	A*26:01	0.063	32	−2.74	8.366	−0.947	0.343476	1
	A*30:02	0.065	28	−0.54	8.752	−0.183	0.855165	1
	A*33:01	0.138	62	2.557	18.996	0.587	0.557473	1
	A*68:01	0.05	21	2.95	7.209	1.099	0.27191	1
	A*68:02	0.052	27	3.18	8.352	1.1	0.271184	1
*HLA_B*	B*07:02	0.067	33	2.66	10.335	0.827	0.407994	1
	B*08:01	0.069	41	−12.18	11.786	−3.548	0.000388	0.01
	B*35:01	0.133	51	6.77	17.467	1.62	0.105266	1
	B*15:03	0.071	35	−0.14	12.033	−0.04	0.967807	1
	B*53:01	0.123	48	9.62	16.341	2.38	0.017324	0.49
	B*58:01	0.075	40	2.28	12.467	0.646	0.518451	1
	B*78:01	0.056	29	−0.53	8.268	−0.184	0.853761	1
*HLA_C*	C*02:02	0.114	53	−0.29	16.851	−0.071	0.943679	1
	C*03:04	0.083	45	−11.888	13.791	−3.201	0.001369	0.04
	C*04:01	0.186	66	11.629	23.828	2.382	0.017205	0.49
	C*06:02	0.089	33	5.52	10.042	1.742	0.081515	1
	C*07:01	0.114	55	−2.403	17.979	−0.567	0.570849	1
	C*16:01/02	0.12	52	−7	14.981	−1.809	0.070526	1
	C*17:01	0.054	27	2.834	7.569	1.03	0.302871	1
HLA-A3/11	No KIR epitope	0.772	81	−2.41	31.893	−0.427	0.669564	1
	Bw4_80I	0.183	69	−0.06	26.511	−0.012	0.990703	1
HLA-Bw4/Bw6	Bw6	0.625	108	−3.907	42.358	−0.6	0.548333	1
	Bw4_80I	0.318	101	3.867	41.641	0.599	0.549034	1
	Bw4_80T	0.056	28	0.04	10.116	0.013	0.989966	1
HLA-C1C2	C2	0.499	99	23.08	40.6	3.622	0.000292	0.008

*Named alleles may indicate the first allele identifier in a longer string of related alleles, but these have been shortened for ease of reading. Full details can be found in table S1.

### Linkage disequilibrium

Pairwise linkage disequilibrium data (LD) for the KIR genes were calculated (figure S2). Contrary to data from other studied human populations [58], [60], [61] and consistent with other findings within Africa [47], we observed reduced LD between KIR genes. We did not identify any pairs of KIR genes that were in perfect LD (r^2^ = 1 : only two of the four possible haplotypes observed), although a number of KIR genes were found to be in complete LD (D′ = 1 : only three of the four possible haplotypes observed). The extent of LD was insufficient for high confidence imputation of missing KIR genotypes for use in FBAT [58].

### Association tests

Any HLA alleles and KIR epitopes with estimated frequencies above 0.05 were included in the FBAT. Three sets of HLA alleles were significantly associated with trachomatous scarring (Table 2). These were HLA-B*08:01 (Z = −3.548, p = 0.0004, corrected p = 0.01), HLA-C*03:04 (Z = −3.201, p = 0.0014, corrected p = 0.04) and the KIR epitope HLA-C1/C2 (Z = 3.622, p = 0.0003, corrected p = 0.008). Only HLA-C1/C2 remained significant (HLA-C2, OR = 1.684 p = 0.0033) in a multivariate case/pseudo-control, additive model that included all three factors (Table 3), indicating that the HLA-C1/C2 epitope was the only significant independent factor of the HLA system that was associated with trachomatous scarring. In line with previous study designs and analyses we divided the data into several subsets [58], [59]. We identified that in the majority of subsets, as with the unselected sample, the relative risk of scarring increased with the number of genomic copies of the HLA-C2 epitope in an additive manner (Table 4). The association of the HLA-C2 homozygote genotype with trachomatous scarring was restricted to the subsets of offspring who were *KIR2DL2* ^+^ and *KIR2DL3* ^+^ (Cen-AB) (OR = 5.95, p = 0.0025, BH corrected p = 0.006) and to those who were *KIR3DL1* ^+^ *KIR3DS1* ^−^ and *KIR2DS1^−^* (Tel-AA) (HLA-C2 homozygote OR = 4.89, p = 0.00006, BH corrected p 0.0004). Elevated odds ratios were observed in sensitivity analyses (Table 4) in F_1_ samples where the case definition was restricted to those with moderate or severe (WHO FPC grade C2 or C3) rather than evidence of any (C1, C2 or C3) scarring.

**Table 3:** Significant results of case/pseudo-control CLR analysis of total family data set.

	Allele	Odds Ratio	P Value
Multivariate CLR TEST	HLA-B*08:01/…*	0.694	0.7000
	HLA-C*03:04/…**	0.500	0.1500
	*HLA-C* EPITOPE C2	1.684	0.0033

Likelihood ratio test = 23.1 on 3 df, p = 0.0000379 n = 580, number of informative events = 152.

**Table 4:** Subset analysis of HLA-C1C2 genotype associations with scarring.

Offspring Genotype	Genotype test HLA-C1/C2	BH Corrected P	Genotype test HLA-C2/C2	BH Corrected P	n	Number of events
Unselected	OR = 2.29 p = 0.0026	0.0061	OR = 3.97 p = 0.000051	0.0004	636	159
*KIR2DL2* ^−^ *KIR2DL3* ^+^ (Cen-AA)	OR = 1.94 p = 0.08	0.120	OR = 2.00 p = 0.15	0.191	296	74
*KIR2DL2* ^+^ *KIR2DL3* ^+^ (Cen-AB)	OR = 2.33 p = 0.057	0.100	OR = 5.95 p = 0.0025	0.006	240	60
*KIR2DL2* ^+^ *KIR2DL3* ^−^ (Cen-BB)	OR = 1.5 p = 0.73	0.786	OR = 6.00 p = 0.13	0.182	76	19
*KIR3DL1* ^+^ *KIR3DS1* ^−^ *KIR2DS1* ^−^ (Tel-AA)	OR = 2.86 p = 0.0013	0.006	OR = 4.89 p = 0.00006	0.0004	524	131
*KIR3DS1* ^+^ and/or *KIR2DS1* ^+^ (Tel-Bx)	OR = 0.52 p = 0.29	0.338	OR = 0.90 p = 0.89	0.890	88	22
Affected cases defined by more severe scarring (WHO FPC score C2 or C3) & *KIR2DL2* ^+^ *KIR2DL3* ^+^ (Cen-AB)	OR = 2.07 p = 0.026	0.052	OR = 3.57 p = 0.0017	0.006	444	111

### Comparison of Gambian KIR gene frequencies to other human populations

**Figure 3:**
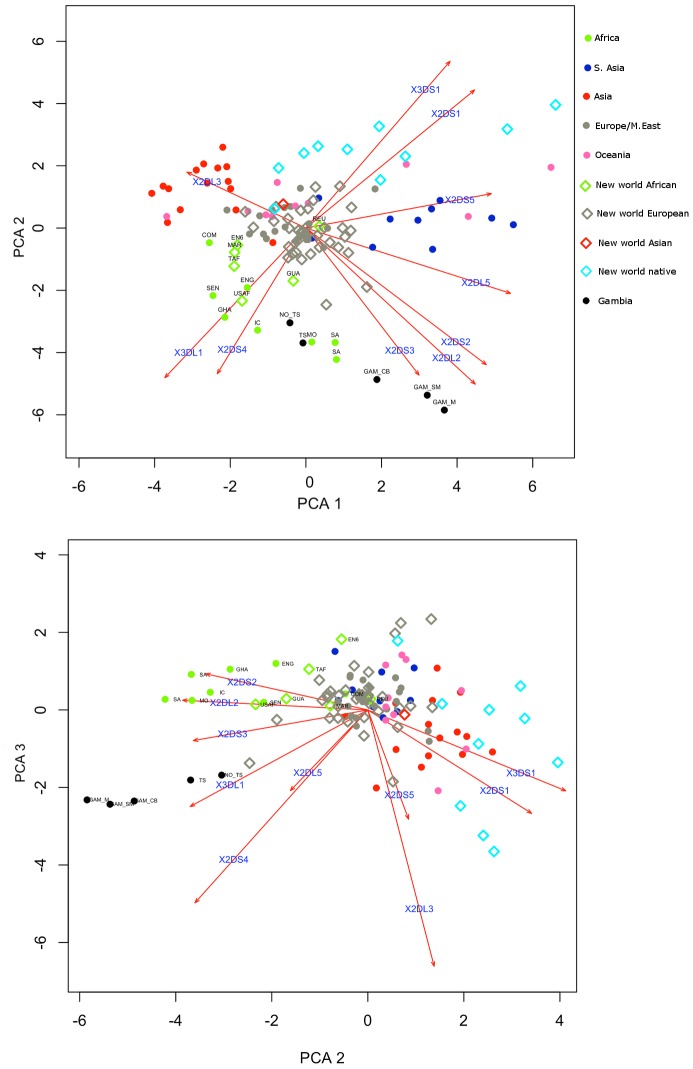
Principle Components Analysis of Gambian KIR frequencies and other world populations. African populations are characterised by high frequencies of the Cen-B (*KIR2DS2*∼*KIR2DL2*, *KIR2DL2*) and Tel-A (*KIR3DS1*∼*KIR2DS4*) haplotypes. Gambian samples, including the P_o_ specimens and malaria cases cluster together and have some of the highest observed frequencies of Cen-B and Tel-A. The proportions of the total variance explained by the first three principal components were respectively 0.42, 0.28 and 0.11. Footnote to figure 3. NO_TS : Parents from Gambian trachoma families, unaffected, TS : Parents from Gambian trachoma families, affected, GAM_SM : Gambian severe malaria cases, GAM_M : Gambian uncomplicated Malaria cases, GCB : Gambian cord bloods, COM : Comoros, ENG : Equatorial New Guinea, GHA : Ghana, IC : Ivory Coast, MO : Morocco, SEN : Senegal, SA : South Africa, EN6 : England – West Midlands Afro-Caribbean, GUA : Guadaloupe, MAR : Martinique, REU : Reunion, TAF (Trinidad Africans), USAF : USA Californian African Americans.

We used Principle Components Analysis (PCA) to compare the KIR gene frequencies observed in the P_0_ generation of the Gambian trachoma families to those observed in other populations where data was available (allelefrequencies.net database, (Figure 3)). The proportions of the total variance explained by the first three principle components were 0.42 (σ = 2.05), 0.28 (σ = 1.69) and 0.11 (σ = 1.03). The P_0_ specimens clustered with other populations of African descent, which could be recognised by the observation of high frequencies of the genes defining the Cen-B (*KIR2DS2*, *KIR2DL2*) and Tel-A (*KIR3DL1* and *KIR2DS4*) haplotypes.

## Discussion

We identified three factors of the HLA system (HLA-C1/C2, HLA-B*08:01 and HLA-C*03:04) that associated with trachomatous scarring. However, the protective associations of HLA-B*08:01 and HLA-C*03:04 observed by univariate analysis were not independent of the HLA-C1/C2 association under a multivariate model. This dependence can be explained by the presence of a common HLA-B*08∼C*03 haplotype, which we estimate to have a frequency of around 5.7% in the Gambian trachoma families. In Senegalese Mandinka, this haplotype has an estimated population frequency of 5.5% [62] whilst in African-Americans the frequency is estimated to be as high as 22% [63]. The associations that were observed between HLA-B*08 and HLA-C*03 (an HLA-C1 group allele) appear to be proxies for the association of the HLA-C1/C2 epitope, which reached statistical significance in univariate analysis because of their high population frequencies and the relatively large contribution they therefore made to the HLA-C epitope data.

We observed patterns of transmission disequilibrium in our sample of families that suggest HLA and KIR genotypes associate with high magnitude increases in the relative risk of scarring in trachomatous disease. Through sensitivity analysis (Table 4) we demonstrated the robustness of the analysis and consistency of the results when the cases were defined by more stringently defined phenotypes relating to severity of scarring.

### HLA-C2 epitopes potentially impair NK cell responses and facilitate chronic Ct infection

The chlamydial protease, CPAF has been reported to interfere with the surface presentation of HLA class I molecules [14], [64]–[66], but recently this has been called in to question by Chen et al. [67]. Kägebein et al. [68] then demonstrated that *Ct* infection does not lead to alteration in normal MHC Class-I expression, maturation or surface presentation. This implies that *Ct* infected cells are unlikely to be targets for missing-self reactions mediated by NK cells which selectively monitor down-regulation or loss of self-type MHC class I on target cells. Instead it is more likely that cytotoxic NK responses in chlamydial infections are controlled by dynamic changes in the expression levels of activating NK receptors. These changes may occur as a result of infection and other environmental triggers [69], [70] and might overwhelm the inhibitory effects of the more strictly expression-regulated [69] NK inhibitory pathways.

HLA-C1:KIR2DL3 inhibited NK cells have weaker inhibitory signals than other HLA-C inhibited cells [29] and may have a lower threshold for activation. Khakoo *et al.* [40] reported that the HLA-C1/C1 *KIR2DL3*/*2DL3* genotype constellation increased probability of clearance of early stage Hepatitis C Virus (HCV) infections. Ahlenstiel *et al.* [42] provided evidence that HLA-C1 homozygotes might be better able to challenge early infections by showing that the proportion of the total NK cell repertoire that is educated and inhibited by HLA-C is ∼50% greater in this group than that in HLA-C2 homozygotes [42]. The same study showed that HLA-C1 inhibited NK cells are better able to mount rapid, intense responses to infection through degranulation and IFNγ secretion [42]. In *Ct* infections, HLA-C1/C1 individuals may be able to limit chronicity by controlling the early stages of *Ct* infections with an NK response that is easily activated, and involves a more substantial component of the NK repertoire than in HLA-C2/C2 individuals. This may also be true of HLA-C2^+^ individuals who possess only weakly responsive KIR2DL1 alleles, such as those alleles that are found on the commonest B haplotypes in Caucasian populations [41]. However, in the Ga-Adangbe population of Ghana, there was a great diversity B haplotypes, none of which were found at high frequency and many of which carried non-attentuated KIR2DL1 alleles [38]. Any assumption about how the presence of Cen-B might indicate reduced cellular inhibition in Gambians should therefore be made with some caution.

### A role for within-person inhibitory KIR diversity in influencing immunopathology

The role of KIR in mediating NK cytotoxic responses is well studied, but it is now clear that KIR expressing NK cells are also a major source of IFNγ [71]. The ability of NK cells to produce IFNγ in response to microbial stimuli is related to the density of NCAM-1 (CD56) expressed on their surface, their KIR genotype and the degree of stimulus by accessory cells. An indication of the strength of regulation imposed by the KIR genotype can be estimated as a ratio, known as the ‘DIM factor’, between the response of the CD56^dim^ (KIR-HLA dependent) and CD56^bright^ (KIR-HLA independent) IFNγ responding populations [71]. The majority of human NK cells in the periphery are CD56^dim^, express KIR and are susceptible to inhibition through KIR-HLA interaction. KIR genotype directly influences the DIM factor, but the exact genotypic conformation that defines this has yet to be elucidated. It has been proposed that the NK cell IFNγ response will be higher in individuals with more KIR educated NK cells, a situation found when there is a greater diversity of within-person inhibitory KIR genes. Experimentally, IFNγ production in CD56^dim^ NK cells showed least inhibition (and the highest DIM factor) in KIR AB heterozygotes [71]. In HLA-C2 homozygotes, we observed a significant *KIR2DL2/L3* heterozygote (Cen-AB) disadvantage (Table 4) and an increased relative risk in those with the Tel-AA genotype. The number of persons with Tel-B genotypes was very low in this study, which reflects the low diversity in the Tel region that was reported in another West African population [38]. The high phenotypic frequency of *KIR3DL1* (Tel-A) in this Gambian population (∼99.6%) indicates that most individuals with the Cen-AB genotype possess at least one Tel-A haplotype. The Cen-AB, Tel-A^+^ genotype represents a full complement of the known MHC specific inhibitory KIRs (*KIR2DL1*, *KIR2DL2*, *KIR2DL3*, *KIR3DL1* and *KIR3DL2*) and this genotype might define a high DIM factor [71]. NK cell clones with a Cen-AB genotype would therefore be relatively resistant to inhibition (DIM factor >1) and would retain the potential for high IFNγ production.

### Common tropical infectious diseases drive selection of high frequencies of trachoma risk genotype constellations

The KIR system exhibits extensive diversity in African populations [47], [72], [73] possibly driven by a high burden of life threatening infectious diseases, that have exerted strong (diversifying) selective pressures on each population [46], [47], [74]. The high prevalence of *Ct* STIs in some African populations has been implicated as a contributory factor to the high incidences of infection related infertility that are observed in Africa [75]. It is therefore surprising that *Ct* disease associated KIR and HLA genotypes are enriched in Africa. One explanation is that opposing selection pressures from other infectious diseases negate selection by *Ct*. Our sample was selected based on disease phenotype and we found KIR gene frequencies similar to other African populations (Figure 3). The Gambian samples are clearly separated from those in other geographical regions by high frequencies of the genes that define the Cen-B and Tel-A haplotypes (Figure 3) [72]. The frequency of HLA-C2 epitopes is reported to be higher in African populations than in other populations [46], [76], [77] and the HLA-C epitope frequencies that we observed are similar to those previously described [77]. Yindom *et al.* [78] reported that the proportion of persons with the constellation HLA-C1 and *KIR2DL2/KIR2DS2* (Cen-B) is higher in cases of malaria than in population matched, cord-blood controls [78]. In a study of a South-East Asian population, Hirayasu *et al.* [79] reported that natural selection may have reduced the frequency of the HLA-C1 and *KIR2DL3* (Cen-A) because this genotype associates with cases of cerebral malaria. Both studies identify HLA-C1 in association with malarial disease, but they implicate different KIR Cen haplotypes. In the Gambian trachoma families, we observed that many Cen-B haplotypes lacked *KIR2DS2*, whilst maintaining *KIR2DL2*. This genotype has previously been identified in an African population [73] and its presence could be explained if *KIR2DS2*, rather than *KIR2DL2*, were mediating the Cen-B risk effect. The combined evidence of several TB studies shows that KIR Cen-A [80]–[82] and Tel-B [82], [83] haplotypes associate with TB cases. We therefore suggest that the HLA-C2 homozygous, Cen-AB, Tel-A^+^ population are more resistant to the complications of both malaria and TB, but more susceptible to trachomatous scarring and that trachomatous scarring (and possibly reduced fertility) is the penalty of increased survival.

### A dual role for KIR-HLA restricted NK cells in trachoma and its implications for chlamydial vaccine development

**Figure 4:**
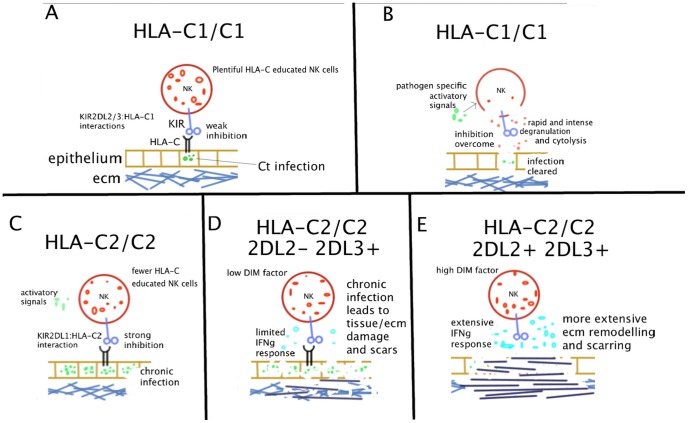
Model of NK cell mediated scarring in trachoma. (A) An HLA-C1/C1 NK cell interacts with HLA class I molecules on a *Ct* infected epithelial cell via interactions between *KIR2DL2/L3* and HLA-C1. HLA-C educated NK cells represent a greater proportion of the total NK repertoire in HLA-C1 homozygotes, compared to those who carry HLA-C2 (B) The inhibitory signals are overcome by loss of the inhibitory HLA-C molecule and/or by activatory signals, which may be from intracellular or extracellular pathogen derived moieties. The NK cytotoxic response is triggered and the target cell is lysed, which culminates in effective resolution of the infection. (C) An HLA-C2/C2 NK cell interacts with MHC class I molecules on *Ct* infected epithelial cell via interactions between KIR2DL1 and HLA-C2. A smaller proportion of the NK cell repertoire is HLA-C educated in this setting. Cytotoxic and IFNγ responses are less likely to be triggered and responses are less intense than in HLA-C1/C1 individuals. Chronic infection is established (D) *KIR2DL2* ^−^, *KIR2DL3* ^+^ individuals have a low DIM factor and NK cell release of IFNγ is more limited. Chronic infection leads to some damage to epithelium and extracellular matrix (ECM) but anti-fibrotic properties of IFNγ are maintained. (E) *KIR2DL2/KIR2DL3* heterozygous individuals have a high DIM factor and NK cells release larger quantities of IFNγ. Chronic infection is coupled with pathologically high levels of IFNγ. HLA-C1/C2 genotypes confer intermediate risks that do not significantly differ from the homozygous individuals. Relative risk of scarring was increased in *KIR2DL2* homozygotes, but this was not significant.

We identified KIR-HLA interactions as an important contributory factor to risk of scarring. The HLA-C2 homozygous, *KIR2DL2* ^+^, *KIR2DL3* ^+^ genotype associates with high relative risk of scarring. We suggest a model that may explain the data in which HLA-C2 may favour chronic infection, whilst *KIR2DL2/L3* heterozygosity favours chronic inflammation (Figure 4). In some aspects this is similar to the model put forward by Hollenbach *et al.* to explain the observation of HLA-C1/C1, *KIR2DL2/L3* heterozygote risk in Crohn's disease [84], which like trachoma is characterised by chronic inflammation and fibrotic immunopathology. It is possible that the high burden of trachomatous scarring, TT and infection related infertility in observed in Africa can be explained in part by unusually high frequencies of HLA-C2 and *KIR2DL2/L3* heterozygosity and the effects of NK cell responsiveness. The therapeutic consequences of such a theory would impact on vaccine immune-therapies and we would expect that current efforts in the development of chlamydial vaccines, adjuvants and immunisation schedules would additionally monitor the boosting or modulating effects on the NK cell compartment. As early as the 1990 it was shown that vaccination with influenza virus was able to elicit NK cell responses [85]. More recent work has demonstrated that many vaccination regimes against viruses boost not only adaptive T and B cell response but also lead to repetitive expansions of NK cells [86]–[88]. Some immunologists have termed these “memory-like NK cell responses” and have now begun to consider the role of these responses in vaccine induced immunity [89]. The effectiveness of NK cells as targets of vaccine immuno-therapy has been described in oncology [90]. Efforts are now required to investigate the role of NK cells in immunity following vaccination with a wider spectrum of bacterial vectors and in natural immunity to infectious diseases such as trachoma.

## Supporting Information

Figure S1 **PBAT estimates of power in this study to detect genetic associations with trachomatous scarring at a range of allele frequencies and effect magnitudes.**
(TIF)

Figure S2 **Evidence for (a) complete [D′ = 1] but (b) not perfect [R^2^ = 1] linkage disequilibrium between pairs of KIR genes.** LD was insufficiently strong to be used to reconstruct missing genotype data in the family study. In (a) dark grey indicates strong evidence of linkage, light grey is uninformative and white indicates strong evidence of recombination. D′ values below 1 are shown. In (b) white indicates R^2^ = 0, shades of grey indicate 0<R^2^<1 and black indicates R^2^ = 1. R squared values below 1 are shown. 2DS4d22 indicates alleles of *KIR2DS4* carrying a 22 bp deletion. 3DP1V indicates alleles of *KIR3DP1* carrying exon 2.
(TIF)

Table S1 **HLA Allele strings used in FBAT and associated frequencies.** For the purposes of grouping for FBAT and in order to maximise statistical power, individual HLA genotypes were assigned to the ‘identifier’ groups if they possessed any or all of the alleles shown in the full genotype string.
(DOCX)
