# Supplementary figures and images for: Conjunctival Scarring in Trachoma Is Associated with the HLA-C Ligand of KIR and Is Exacerbated by Heterozygosity at KIR2DL2/KIR2DL3
Source: PLoS Negl Trop Dis. 2014 Mar 20;8(3):e2744. doi: 10.1371/journal.pntd.0002744 (PMC3961204; doi:10.1371/journal.pntd.0002744)

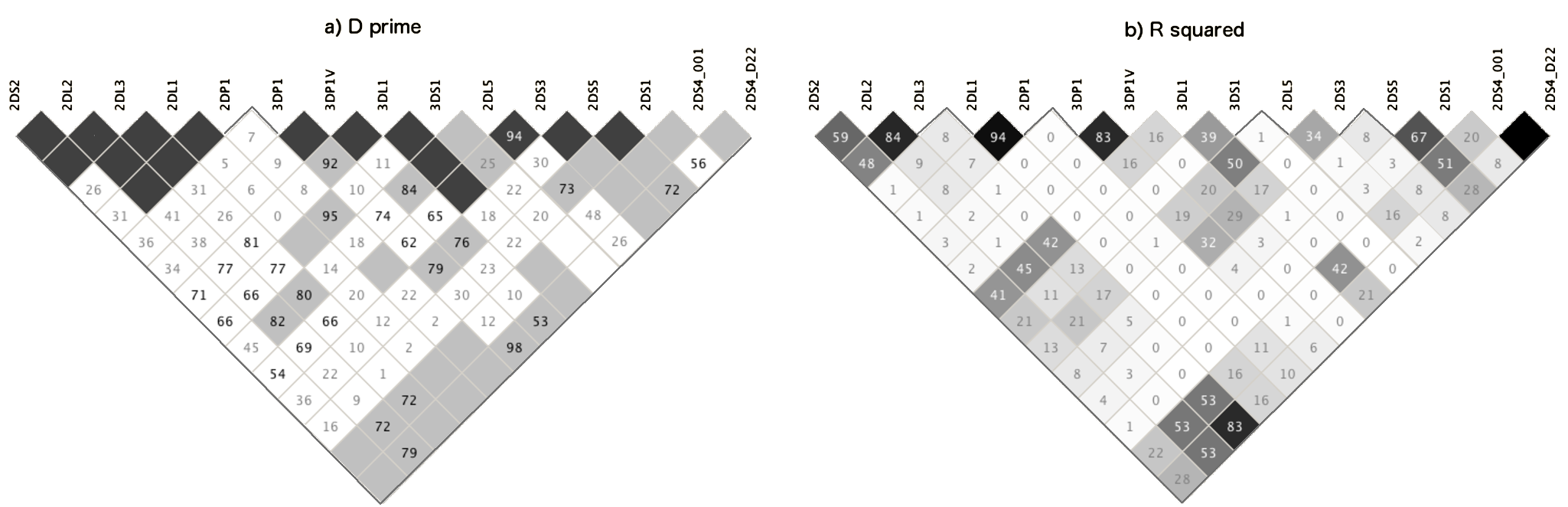

Supplement: Figure S2 — Evidence for (a) complete [D′ = 1] but (b) not perfect [R2 = 1] linkage disequilibrium between pairs of KIR genes. LD was insufficiently strong to be used to reconstruct missing genotype data in the family study. In (a) dark grey indicates strong evidence of linkage, light grey is uninformative and white indicates strong evidence of recombination. D′ values below 1 are shown. In (b) white indicates R2 = 0, shades of grey indicate 0<R2<1 and black indicates R2 = 1. R squared values below 1 are shown. 2DS4d22 indicates alleles of KIR2DS4 carrying a 22 bp deletion. 3DP1V indicates alleles of KIR3DP1 carrying exon 2. (TIF) [file pntd.0002744.s002.tif]
