# Supplementary material for: Conjunctival Scarring in Trachoma Is Associated with the HLA-C Ligand of KIR and Is Exacerbated by Heterozygosity at KIR2DL2/KIR2DL3
Source: PLoS Negl Trop Dis. 2014 Mar 20;8(3):e2744. doi: 10.1371/journal.pntd.0002744 (PMC3961204; doi:10.1371/journal.pntd.0002744)
Supplement: Table S1 — HLA Allele strings used in FBAT and associated frequencies. For the purposes of grouping for FBAT and in order to maximise statistical power, individual HLA genotypes were assigned to the ‘identifier’ groups if they possessed any or all of the alleles shown in the full genotype string. (DOCX) [file pntd.0002744.s003.docx]

Table S1 : HLA Allele strings used in FBAT and associated frequencies. For the purposes of grouping for FBAT and in order to maximise statistical power, individual HLA genotypes were assigned to the ‘identifier’ groups if they possessed any or all of the alleles shown in the full genotype string.

| Identifier | Frequency | Full Genotype string |
| --- | --- | --- |
| HLA-A*01:01 | 0.040 | 01:01/01:02/01:04N/01:06/01:09/01:14/01:16N/01:17/01:18N/01:22N/01:23/01:24/01:25/01:29/01:30/01:31N/01:32/01:33/01:34N/01:35/01:36/01:37/01:38/01:39/01:40/01:41/01:42/01:44/01:45/01:47/01:50 |
| HLA-A*01:02 | 0.025 | 01:02/01:24/01:43 |
| HLA-A*02:01 | 0.067 | 02:01/02:04/02:07/02:09/02:105/02:107/02:109/02:110/02:111/02:113/02:114/02:116/02:118/02:119/02:120/02:121/02:123/02:125/02:132/02:133/02:134/02:138/02:139/02:140/02:141/02:145/02:147/02:149/02:150/02:151/02:153/02:156/02:159/02:15N/02:160/02:161/02:162/02:164/02:165/02:166/02:167/02:168/02:17/02:173/02:175/02:176/02:177/02:18/02:181/02:182/02:185/02:187/02:188/02:189/02:192/02:193/02:196/02:197/02:199/02:20/02:24/02:25/02:29/02:30/02:31/02:32N/02:33/02:34/02:42/02:43N/02:49/02:53N/02:59/02:60/02:62/02:64/02:66/02:67/02:68/02:70/02:71/02:73/02:74/02:75/02:76/02:77/02:82N/02:83N/02:85/02:86/02:88N/02:89/02:92/02:93/02:94N/02:95/02:96/02:97 |
| HLA-A*02:02 | 0.048 | 02:02/02:102/02:115/02:155/02:186/02:47/02:63 |
| HLA-A*02:05 | 0.019 | 02:05/02:08/02:172/02:179 |
| HLA-A*03:01 | 0.040 | 03:01/03:03N/03:04/03:07/03:08/03:11N/03:12/03:13/03:14/03:15/03:17/03:20/03:21N/03:22/03:25/03:26/03:27/03:28/03:29/03:33/03:34/03:35/03:37/03:38/03:39/03:44/03:45/03:46/03:47/03:48/03:49/03:51/03:52/03:53/03:55/03:56/03:58/03:60/03:61/03:62/03:63/03:66/03:67/03:68N/03:69N/03:71 |
| HLA-A*23:01 | 0.164 | 23:01/23:03/23:04/23:05/23:06/23:07N/23:08N/23:09/23:10/23:11N/23:12/23:13/23:14/23:15/23:16/23:17/23:18/23:19Q/23:20/23:21/23:22/23:23/23:24/23:25 |
| HLA-A*24:02 | 0.015 | 24:02/24:09N/24:11N/24:15/24:17/24:20/24:21/24:25/24:26/24:27/24:30/24:34/24:35/24:36N/24:37/24:38/24:39/24:40N/24:41/24:43/24:45N/24:47/24:48N/24:49/24:50/24:51/24:52/24:53/24:58/24:59/24:63/24:64/24:68/24:69/24:70/24:72/24:73/24:74/24:76/24:78/24:79/24:80/24:81/24:83N/24:84N/24:85/24:86N/24:90N/24:93/24:95/24:96/24:98 |
| HLA-A*26:01 | 0.063 | 26:01/26:02/26:04/26:09/26:10/26:11N/26:12/26:15/26:16/26:17/26:22/26:23/26:24/26:25N/26:26/26:27/26:28/26:31/26:32/26:33/26:35/26:36/26:37/26:38/26:39/26:41/26:42 |
| HLA-A*29:01 | 0.037 | 29:01/29:02/29:03/29:04/29:06/29:07/29:08N/29:10/29:11/29:12/29:15/29:16/29:17 |
| HLA-A*30:01 | 0.046 | 30:01/30:01:01/30:01:02/30:11/30:14L/30:15/30:18/30:19/30:20/30:23/30:24/30:26/30:31 |
| HLA-A*30:02 | 0.067 | 30:02/30:03/30:07/30:10/30:12/30:25/30:27N/30:28/30:32/30:33/30:34 |
| HLA-A*31:01 | 0.006 | 31:01/31:09/31:11/31:12/31:13/31:14N/31:15/31:16/31:18/31:19/31:20/31:23/31:26/31:27/31:28 |
| HLA-A*32:01 | 0.033 | 32:01/32:05/32:06/32:07/32:08/32:09/32:11Q/32:12/32:13/32:14/32:16/32:17/32:18/32:21 |
| HLA-A*33:01 | 0.138 | 33:01/33:03/33:04/33:05/33:06/33:07/33:10/33:12/33:14/33:15/33:16/33:17/33:18/33:20/33:21/33:23/33:25/33:26/33:27/33:29 |
| HLA-A*34:02 | 0.033 | 34:02/34:04/34:07 |
| HLA-A*66:01 | 0.010 | 66:01/66:04/66:05/66:08/66:12 |
| HLA-A*66:02 | 0.006 | 66:02/66:03 |
| HLA-A*68:01 | 0.046 | 68:01/68:08/68:10/68:13/68:14/68:22/68:25/68:27/68:35/68:43/68:45 |
| HLA-A*68:02 | 0.052 | 68:02/68:18N/68:28/68:31/68:34/68:39/68:40/68:44 |
| HLA-A*74:01 | 0.040 | 74:01/74:02/74:03/74:06/74:07/74:08/74:09/74:11/74:12N/74:14N |
| HLA-A*80:01 | 0.004 | 80:01 |
|  |  |  |
| HLA-B*07:02 | 0.062 | 07:02/07:05/07:06/07:09/07:10/07:11/07:12/07:18/07:21/07:22/07:23/07:26/07:30/07:35/07:39/07:41/07:44/07:45/07:46/07:49N/07:51/07:52/07:57/07:58/07:59/07:61/07:62/07:67N/07:68/07:73/07:74/07:75/07:76/07:80/07:82/07:83/07:87/07:88/07:89/07:90/07:92/07:93/07:97/07:98/07:99 |
| HLA-B*08:01 | 0.079 | 08:01/08:03/08:05/08:08N/08:10/08:11/08:12/08:15/08:16/08:18/08:19N/08:22/08:23/08:24/08:27/08:28/08:29/08:30N/08:31/08:33/08:34/08:35/08:36/08:39/08:41/08:44/08:45/08:46/08:47/08:48/08:51/08:54 |
| HLA-B*13:02 | 0.004 | 13:02/13:08Q |
| HLA-B*14:02 | 0.029 | 14:02/14:09/14:16/14:17 |
| HLA-B*15:03 | 0.069 | 15:03/15:103/15:123/15:127/15:132/15:151/15:156/15:158/15:173/15:61/15:62/15:74/15:98 |
| HLA-B*15:10 | 0.042 | 15:10/15:134/15:18/15:72/15:90 |
| HLA-B*15:16 | 0.025 | 15:16/15:67/15:95 |
| HLA-B*15:18 | 0.002 | 15:18/15:72 |
| HLA-B*18:01 | 0.031 | 18:01/18:02/18:03/18:05/18:06/18:07/18:08/18:11/18:17N/18:20/18:22/18:23N/18:24/18:25/18:27/18:28/18:31/18:32/18:34/18:36/18:38/18:39/18:40/18:41/18:42/18:44/18:45/18:46/18:47 |
| HLA-B*27:03 | 0.023 | 27:03/27:05/27:13/27:17/27:32/27:37/27:38/27:45/27:48/27:55/27:56/27:58/27:60 |
| HLA-B*35:01 | 0.129 | 35:01/35:04/35:05/35:07/35:09/35:11/35:17/35:19/35:24/35:27/35:29/35:30/35:32/35:37/35:40N/35:41/35:42/35:50/35:52/35:53N/35:54/35:57/35:64/35:68/35:77/35:78/35:89/35:90/35:91/35:92/35:94/35:97 |
| HLA-B*37:01 | 0.008 | 37:01/37:03N/37:06/37:15/37:17/37:18/37:20 |
| HLA-B*39:10 | 0.004 | 39:10/39:20 |
| HLA-B*40:02 | 0.006 | 40:02/40:29/40:35/40:37/40:50/40:56/40:57/40:64/40:78/40:82/40:85/40:89/40:90/40:91/40:94 |
| HLA-B*41:02 | 0.010 | 41:02/41:11 |
| HLA-B*41:03 | 0.008 | 41:03 |
| HLA-B*42:01 | 0.037 | 42:01/42:02/42:08/42:10 |
| HLA-B*44:02 | 0.013 | 44:02/44:03/44:13/44:26/44:28/44:30/44:32/44:35/44:36/44:37/44:38/44:39/44:50/44:61N/44:65/44:69/44:73/44:85/44:89/44:94/44:98 |
| HLA-B*44:10 | 0.002 | 44:10 |
| HLA-B*45:01 | 0.013 | 45:01/45:03/45:05/45:07/45:09 |
| HLA-B*47:01 | 0.002 | 47:01 |
| HLA-B*49:01 | 0.050 | 49:01/49:04/49:05/49:06/49:08 |
| HLA-B*50:01 | 0.033 | 50:01/50:04/50:08 |
| HLA-B*51:01 | 0.023 | 51:01/51:03/51:04/51:11N/51:14/51:17/51:18/51:24/51:26/51:27N/51:28/51:29/51:30/51:32/51:33/51:35/51:37/51:38/51:46/51:48/51:49/51:51/51:53/51:55/51:58/51:60/51:63/51:65/51:69/51:71/51:75/51:76/51:77/51:80/51:84/51:86 |
| HLA-B*51:02 | 0.002 | 51:02 |
| HLA-B*52:01 | 0.017 | 52:01 |
| HLA-B*53:01 | 0.113 | 53:01/53:02/53:08/53:10/53:14/53:18/53:20 |
| HLA-B*55:01 | 0.004 | 55:01/55:03/55:15/55:25/55:29/55:33 |
| HLA-B*56:01 | 0.012 | 56:01/56:19N/56:20/56:24 |
| HLA-B*57:01 | 0.002 | 57:01/57:03/57:06/57:15/57:17/57:18 |
| HLA-B*57:02 | 0.008 | 57:02 |
| HLA-B*57:04 | 0.004 | 57:04 |
| HLA-B*58:01 | 0.079 | 58:01/58:02/58:04/58:10N/58:11/58:13/58:15/58:16/58:17N/58:18/58:19/58:21/58:23/58:24/58:26 |
| HLA-B*78:01 | 0.056 | 78:01/78:02 |
| HLA-B*82:01 | 0.002 | 82:01 |
|  |  |  |
| HLA-C*01:02 | 0.021 | 01:02/01:03/01:07/01:08/01:11/01:14/01:15/01:16/01:17/01:18/01:19/01:20/01:23/01:25/01:26/01:27/01:29/01:30/01:31/01:32/01:33 |
| HLA-C*02:02 | 0.108 | 02:02/02:02:02/02:02:06/02:04/02:05/02:07/02:08/02:09/02:10/02:11/02:12/02:14/02:15/02:19/02:20/02:21/02:23/02:24/02:25Q/02:27/02:28/02:29/02:31 |
| HLA-C*03:02 | 0.052 | 03:02/03:05/03:15/03:17/03:25/03:27/03:33/03:35/03:36/03:60/03:71 |
| HLA-C*03:03 | 0.010 | 03:03/03:04/03:06/03:09/03:11/03:12/03:19/03:20N/03:22Q/03:23/03:24/03:26/03:30/03:31/03:32/03:37/03:39/03:40/03:42/03:43/03:44/03:46/03:48/03:50/03:52/03:53/03:54/03:56/03:57/03:59/03:62/03:63/03:64/03:66/03:68/03:69/03:72/03:73/03:74/03:75/03:77/03:7803:04/03:06/03:07/03:09/03:19/03:23/03:24/03:26/03:28/03:32/03:37/03:38/03:44/03:45/03:46/03:48 |
| HLA-C*03:04 | 0.092 | 03:04/03:06/03:07/03:08/03:09/03:19/03:23/03:24/03:25/03:26/03:27/03:28/03:32/03:35/03:37/03:38/03:40/03:41/03:42/03:44/03:45/03:46/03:47/03:48/03:54/03:57/03:63/03:64/03:65/03:70/03:72/03:73/03:74/03:77/03:7804:01/04:05/04:07/04:09N/04:19/04:20/04:24/04:25/04:27/04:28/04:30/04:31/04:33 |
| HLA-C*04:01 | 0.175 | 04:01/04:04/04:05/04:07/04:09N/04:10/04:11/04:12/04:15/04:17/04:18/04:19/04:20/04:23/04:24/04:25/04:26/04:27/04:28/04:29/04:30/04:31/04:33/04:35/04:36/04:38/04:39/04:40/04:41/04:43/04:44/04:45/04:46/04:47/04:48/04:49/04:50/04:51/04:52/04:53 |
| HLA-C*05:01 | 0.025 | 05:01/05:03/05:05/05:06/05:07N/05:13/05:15/05:16/05:19/05:21/05:22/05:24/05:25/05:28/05:31/05:33/05:34 |
| HLA-C*05:20 | 0.015 | 05:20/05:32 |
| HLA-C*06:02 | 0.079 | 06:02/06:04/06:10/06:11/06:12/06:13/06:14/06:15/06:16N/06:17/06:18/06:19/06:20/06:21/06:22/06:24/06:25/06:26/06:27/06:28/06:29 |
| HLA-C*07:01 | 0.119 | 07:01/07:05/07:06/07:07/07:09/07:16/07:18/07:20/07:21/07:24/07:27/07:30/07:35/07:36/07:40/07:43/07:44/07:52/07:55N/07:57/07:58/07:59/07:65/07:69/07:70/07:71/07:73/07:77/07:78/07:81/07:82/07:83/07:86/07:89/07:91/07:93/07:94/07:95/07:98N |
| HLA-C*07:02 | 0.035 | 07:02/07:03/07:10/07:13/07:25/07:29/07:31/07:32N/07:33N/07:38/07:39/07:42/07:46/07:47/07:48/07:49/07:50/07:51/07:54/07:56/07:61N/07:62/07:64/07:66/07:67/07:72/07:74/07:75/07:76/07:80/07:84/07:87/07:88/07:90/07:97/07:99 |
| HLA-C*07:19 | 0.004 | 07:19 |
| HLA-C*07:28 | 0.002 | 07:28/07:35/07:55N |
| HLA-C*08:02 | 0.004 | 08:02/08:04/08:05/08:07/08:12/08:17/08:19/08:28 |
| HLA-C*12:01 | 0.002 | 12:01/12:02/12:03/12:06/12:13 |
| HLA-C*12:20 | 0.002 | 12:20 |
| HLA-C*14:02 | 0.027 | 14:02/14:04/14:07N/14:08/14:11/14:13/14:14 |
| HLA-C*15:04 | 0.019 | 15:04/15:05/15:06/15:09/15:18/15:19/15:24 |
| HLA-C*16:01 | 0.125 | 16:01/16:02/16:06/16:07/16:08/16:09/16:10/16:11/16:12/16:13/16:14/16:15/16:16/16:17 |
| HLA-C*17:01 | 0.056 | 17:01/17:02/17:03/17:04/17:06 |
| HLA-C*18:01 | 0.008 | 18:01/18:02 |
| HLA-C*05:20/08:02 | 0.019 | 05:20/05:32/08:02/08:04/08:05/08:07/08:12/08:17/08:19/08:28 |
| HLA-C*12:20/16:01 | 0.002 | 12:20/16:01/16:02/16:06/16:07/16:08/16:09/16:10/16:11/16:12/16:13/16:14/16:15/16:16/16:17 |
